# Sensing High 17β-Estradiol Concentrations Using a Planar Microwave Sensor Integrated with a Microfluidic Channel

**DOI:** 10.3390/bios13050541

**Published:** 2023-05-12

**Authors:** Supakorn Harnsoongnoen, Panida Loutchanwoot, Prayook Srivilai

**Affiliations:** 1 The Biomimicry for Sustainable Agriculture, Health, Environment and Energy Research Unit, Department of Physics, Faculty of Science, Mahasarakham University, Kantarawichai District, Maha Sarakham 44150, Thailand; 2 Department of Biology, Faculty of Science, Mahasarakham University, Kantarawichai District, Maha Sarakham 44150, Thailand; prayook@msu.ac.th

**Keywords:** planar microwave sensor, fractal geometry, microfluidic channel, complementary split-ring resonator (CSRR), 17β-estradiol (E2)

## Abstract

The global issue of pollution caused by endocrine-disrupting chemicals (EDCs) has been gaining increasing attention. Among the EDCs of environmental concern, 17β-estradiol (E2) can produce the strongest estrogenic effects when it enters the organism exogenously through various routes and has the potential to cause harm, including malfunctions of the endocrine system and development of growth and reproductive disorders in humans and animals. Additionally, in humans, supraphysiological levels of E2 have been associated with a range of E2-dependent disorders and cancers. To ensure environmental safety and prevent potential risks of E2 to human and animal health, it is crucial to develop rapid, sensitive, low cost and simple approaches for detecting E2 contamination in the environment. A planar microwave sensor for E2 sensing is presented based on the integration of a microstrip transmission line (TL) loaded with a Peano fractal geometry with a narrow slot complementary split-ring resonator (PF-NSCSRR) and a microfluidic channel. The proposed technique offers a wide linear range for detecting E2, ranging from 0.001 to 10 mM, and can achieve high sensitivity with small sample volumes and simple operation methods. The proposed microwave sensor was validated through simulations and empirical measurements within a frequency range of 0.5–3.5 GHz. The E2 solution was delivered to the sensitive area of the sensor device via a microfluidic polydimethylsiloxane (PDMS) channel with an area of 2.7 mm^2^ and sample value of 1.37 µL and measured by a proposed sensor. The injection of E2 into the channel resulted in changes in the transmission coefficient (S_21_) and resonance frequency (F_r_), which can be used as an indicator of E_2_ levels in solution. The maximum quality factor of 114.89 and the maximum sensitivity based on S_21_ and F_r_ at a concentration of 0.01 mM were 1746.98 dB/mM and 40 GHz/mM, respectively. Upon comparing the proposed sensor with the original Peano fractal geometry with complementary split-ring (PF-CSRR) sensors without a narrow slot, several parameters were evaluated, including sensitivity, quality factor, operating frequency, active area, and sample volume. The results showed that the proposed sensor exhibited an increased sensitivity of 6.08% and had a 40.72% higher quality factor, while the operating frequency, active area, and sample volume showed decreases of 1.71%, 25%, and 28.27%, respectively. The materials under tests (MUTs) were analyzed and categorized into groups using principal component analysis (PCA) with a K-mean clustering algorithm. The proposed E2 sensor has a compact size and simple structure that can be easily fabricated with low-cost materials. With the small sample volume requirement, fast measurement with a wide dynamic range, and a simple protocol, this proposed sensor can also be applied to measure high E2 levels in environmental, human, and animal samples.

## 1. Introduction

Nowadays, there is a growing interest in the state of the environment and the impact of pollution on the health of living organisms. Efforts are being made to protect the environment and minimize pollution levels in order to safeguard the well-being of both humans and animals. Sex hormones are considered one of the major pollutants that contaminate the environment. This issue is currently receiving growing attention from the scientific community [1]. The major female sex steroid, 17β-estradiol (E2), is involved in the development and maintenance of many reproductive and nonreproductive tissues, including neuroendocrine tissues [2,3,4]. There are various potential sources of environmental E2 contamination, including animal farms, slaughterhouses, and urban areas with high populations (hormone contraceptives and E2 replacement therapy) [5,6]. When tissues containing E2 and various potential sources of E2 are improperly discarded, including with medical waste or in sewage, they can enter the environment. Once in the environment, E2 can contaminate water sources. This can lead to E2 exposure in both wildlife and humans who rely on these water sources for drinking and other purposes. Elevated levels of E2 have been found to be present in the environment and can potentially have negative impacts on reproductive function and development in both animals and humans. High E2 levels can disrupt the normal functioning of the neuroendocrine and reproductive endocrine systems, which play a critical role in regulating reproductive processes, i.e., disrupting the normal functioning of endogenous sex steroids and disrupting normal sex steroid hormone biosynthesis and metabolism, or altering growth, development, and morphology [3,4,7,8,9,10]. Prior studies have found that high circulating E2 concentrations (>10 pM) are strongly correlated with an increased risk of developing E2-dependent breast cancer [7]. Recent reports have revealed that higher urinary E2 levels—above 0.5 mM—are associated with a reduced risk of breast cancer [8]. E2 concentrations (>9 mM) found in water cause potentially irreversible environmental risks to aquatic ecosystems and animals, such as stimulation of growth, inhibition of gonad growth and development, and suppression of gametogenesis, leading to the extinction of wild fish populations [9,10]. It is evident that there is currently E2 contamination in the environment, particularly in surface water, where concentrations are higher than the normal E2 concentrations found in living organisms. Therefore, subsequent investigations have focused on the development of a simple, fast, and sensitive method that can detect E2 at concentrations higher than those typically detected in living organisms, as well as in aquatic ecosystems. Hence, the suspected effects, i.e., the association between endocrine disruptions and the potential adverse effects on human health, ecologically or economically important animal species, and the environment may have been predicted, prevented, and perhaps remediated up to the present.

E2 can be measured in several ways, including high-performance liquid chromatography-tandem mass spectrometry (HPLC-MS/MS) [11], liquid chromatography with tandem mass spectrometry (LC-MS/MS) [12], and gas chromatography/tandem mass spectrometry (GC-MS/MS) [13]. However, these techniques are time-consuming and complex, involving sample pretreatment, expensive equipment, and well-trained personnel. Recently, sensors have been designed using aptamer-based analytical methods for the detection of E2, such as electrochemical, colorimetric, fluorescent, photoelectrochemical aptasensors, and vertically oriented silicon nanowire (vSiNW) [14,15,16,17,18,19,20,21,22,23]. These sensors detect the concentration of E2 by determining E2 binding using a DNA aptamer bonded to a gold nanoparticle, 3-aminopropyltriethoxysilane (APTES), gold thin film, RhoB, and TiO_2_-BiVO_4_, and then measuring the current (I), wavelength (λ), absorbance (A), and fluorescence intensity (F). All of the methods listed above are aptamer-based techniques, with the sensor exhibiting excellent selectivity for E2 and being able to differentiate it from other EDCs. However, the preparation of these aptasensors is complex and involves a large number of processes and procedures. In addition, large sample volumes are required for measurement, and only a small range of concentrations can be detected.

Microwave sensors are attractive for many reasons, including high sensitivity and fast and cost-effective measurements. Meanwhile, microfluidic technology has the great advantage of being small, with a nanoscale-sample size and fast detection time. Microwave sensors integrated with microfluidics have demonstrated significant benefits in tiny devices, including low cost, non-invasive operation, and ease of use [24]. The integration of microwave sensors with microfluidic channels for measuring MUTs in the liquid phase by detecting the notch magnitude and resonance frequency is altered [25,26,27,28,29,30,31,32,33,34,35]. In liquid MUTs sensing, the sensitive region of various microwave sensor structures is responded to via injecting the MUTs into a microfluidic channel. Any variation in the electrical properties of the MUTs leads to alterations in the magnitude and resonance frequency of the response between the microwave sensor and the MUTs. TL-loaded and split-ring resonators (SRRs) [36] and complement split-ring resonators (CSRRs) integrated with microfluidic channels have recently been designed and investigated [37,38,39,40,41,42,43,44,45,46]. From our literature survey, it is apparent that biosensor devices operating in the microwave frequency band have been proposed for the measurement of various biomolecules, including DNA, streptavidin, and biotin. Furthermore, these sensors have also been proposed for the detection of plasma prostate-specific antigens and cortisol [47,48,49]. Our findings demonstrate the significant potential of microwave sensing techniques in the field of biosensing. However, the fabrication process for these biosensor devices is rather complicated due to the need to immobilize anti-PSA, anti-cortisol, thiol-linked single-stranded DNA, biotin, and streptavidin on the gold surface. These biosensing techniques have a narrow dynamic range, low sensitivity, and require a significant amount of time to analyze. Additionally, they operate at a high frequency, need a large sample volume, and are expensive.

We have found a significant research gap in the field of microwave sensing. Specifically, we found that the detection of highly concentrated E2 reagents dissolved in ethanol using microwave sensors has not been previously studied or proposed. Therefore, our research aimed to address this gap by proposing a novel approach for the detection of E2 reagents using microwave sensing techniques. In this study, we developed a novel sensor by integrating a microwave sensor with a microfluidic channel for E2 detection. This approach is simple and does not require any aptamer or receiver to be immobilized on the gold surface. As a result, our approach enables faster, less expensive, and simplified specimen preparation, identification, and quantification of high E2 concentrations compared with the biosensor devices described earlier. Furthermore, the proposed method achieved higher sensitivity by reducing the slot size of the SRR of the microwave sensor so that there is a higher electric field intensity in that region, which directly affects the sensitivity of the sensor. At the same time, the volume and region of the measurements were limited using a microfluidic channel. We also studied and proposed MUT clustering through a PCA-based data reduction method in conjunction with a K-means clustering algorithm.

## 2. Materials and Methods

### 2.1. Sensor Design

**Figure 1:**
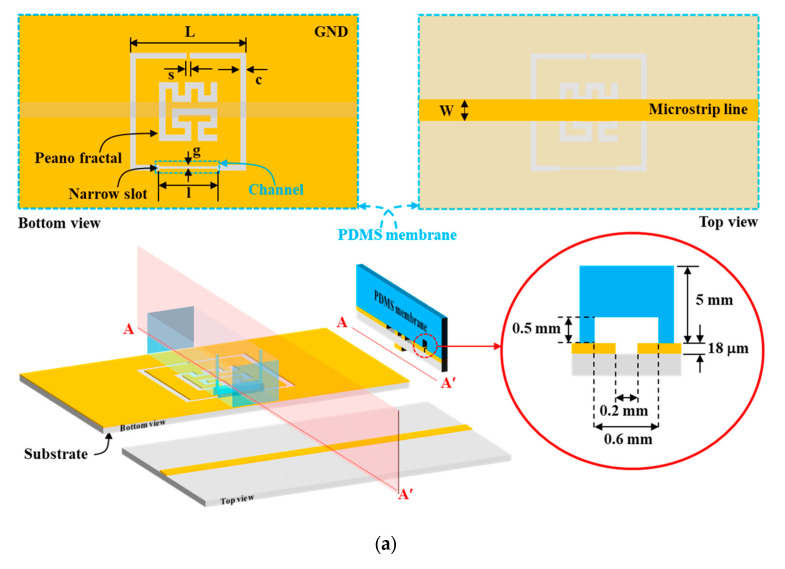

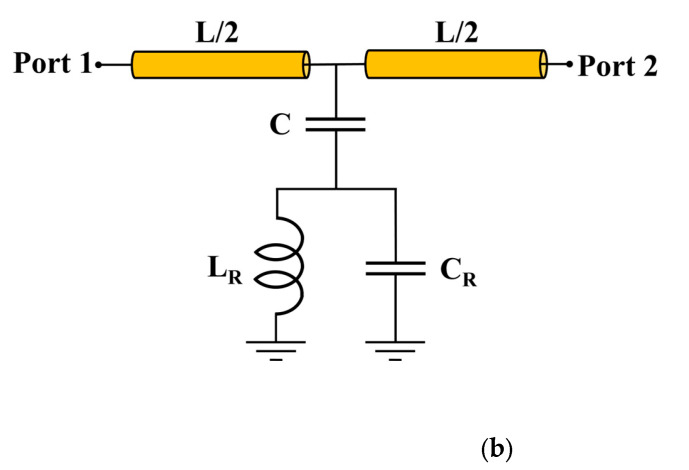
Microfluidic channel with PF-NSCSRR sensor. (**a**) Schematic layout of PF-NSCSRR. The blue dotted line is the region covered by the PDMS membrane, and the cross-section along the AA′ line shows the three-dimensional structure and dimensions of the microfluidic channel, which is shown as a red circular solid line. (**b**) Equivalent circuit model.

Figure 1a shows the schematic layout of PF-NSCSRR and the region covered by the PDMS membrane. The PF-NSCSRR has a planar structure that is etched at the copper ground plane (bottom view) with a load microstrip line on opposite sides of the substrate (top view). A photolithography method with a negative dry film photoresist was used to fabricate the PF-NSCSRR device. A double-sided copper with a substrate of 0.508 mm-thick Rogers RO6002, a dielectric constant of 2.94, and a loss tangent of 0.0012 was used to fabricate the proposed sensor. To measure the dielectric constant and loss tangent of water and ethanol, the microwave sensor was designed to operate within the 1–2.5 GHz frequency range [34,38,39,40,41,42,43]. This frequency range was selected because it is close to the vibrational frequency of water and ethanol molecules, allowing for better interaction with the microwave sensor. In this study, the quantity of E2 dissolved in ethanol was measured using the PF-NSCSRR device, which was designed to operate within the frequency range that responds well to ethanol and water, as described in previous studies [34,38,39,40,41,42,43].

The geometric parameters of the PF-NSCSRR device were designed to operate within the 0.5–3 GHz frequency range through modification of the width and length of CSRR. Table 1 displays the geometrical parameters of the PF-NSCSRR device. The narrow slot width was located along the CSRR’s lower edge, and the size and fractal count of the Peano fractal were centered within the CSRR structure [50,51,52,53,54]. Figure 1b shows the equivalent circuit model demonstration of the proposed sensor; L is the inductance of the transmission line per unit length, C is the coupling capacitance between the microstrip line and the CSRR structure, and C_R_ and L_R_ are the capacitance and inductance of the CSRR, respectively. The resonance frequency (F_r_) was described as follows [55]:

(1) $$F_{r}=\frac{1}{2\pi \sqrt{L_{R}+\left(C+C_{R}\right)}}$$

In this research, the design of the microwave sensor structure was achieved by focusing on making the operating frequency lower compared with conventional structures, while maintaining high electric field strength under the same structural dimensions. Regarding the methods used in this study for modifying the operating frequency and electric field intensity of the microwave sensor, the C and C_R_ values were modified in the equivalent circuit depicted in Figure 1b by etching copper into the middle of the CSRR and narrowing the slot into the position used for sample measurement.

**Figure 2:**
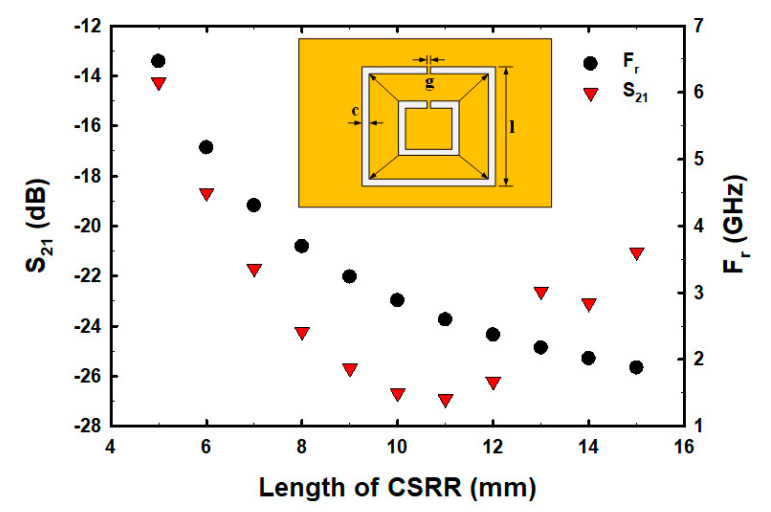
The S_21_ and F_r_ with varied CSRR sizes.

**Figure 3:**
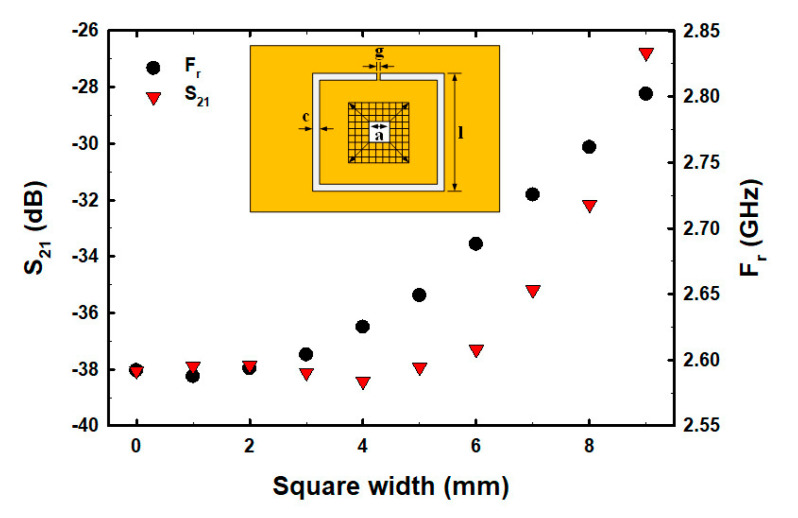
The S_21_ and F_r_ with varied etched square areas (a^2^).

Although the number and centered fractional pattern within the CSRR structure have been partially studied [50,51,52,53,54], the square width of CSRR, the etched square size area in the middle of the ground plane inside CSRR, and the varying fractal geometry centered within the CSRR structure have not been studied and analyzed. In order to determine the optimal size and structure before fabricating the actual parts for E2 measurements, we conducted a simulation to determine the optimal square width of CSRR, etched square size area, and fractal geometry within the ground plane of CSRR. The size of the CSRR structure was determined and simulation results showed that as the square width of CSRR increased, the resonance frequency (F_r_) decreased, whereas the S_21_ decreased within the range of 5–11 mm and then increased (Figure 2). The factorial pixelated complementary split-ring resonators (FPCSRRs) structure in this research was designed and analyzed under a square width of CSRR of 11 mm due to the lowest magnitude of S_21_. The etched square size area was varied in the middle of the ground plane inside CSRR. As the square width increased, the F_r_ increased, whereas the S_21_ decreased within the range of 2–4.5 mm and then increased (Figure 3).

A square of size 4.5 mm × 4.5 mm in the middle CSRR structure of the ground plane was pixilated into 9 × 9 pixels with a resolution of 0.5 mm × 0.5 mm, as shown in Figure 3.

**Figure 4:**
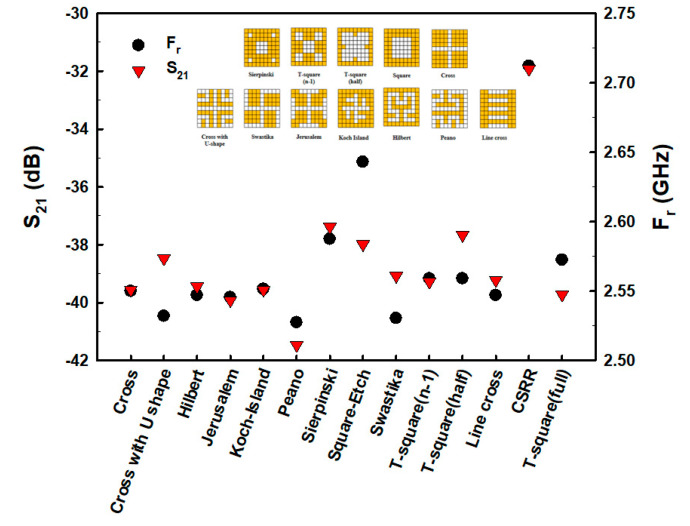
The S_21_ and F_r_ of fractal pixel geometry.

The fractal geometry was then formatted in various forms within the 9 × 9 pixelate area, including Sierpinski, T-square (n − 1), T-square (half), Square, Cross, Cross with U-shape, Swastika, Jerusalem, Koch Island, Hilbert, Peano, and Line cross, as shown in the inserts of Figure 4. The results of the simulation demonstrated that the Peano fractal geometry had the lowest F_r_ and S_21_ values (Figure 4). Therefore, the Peano fractal geometry was chosen for the evaluation of E2 measurements in this study.

**Figure 5:**
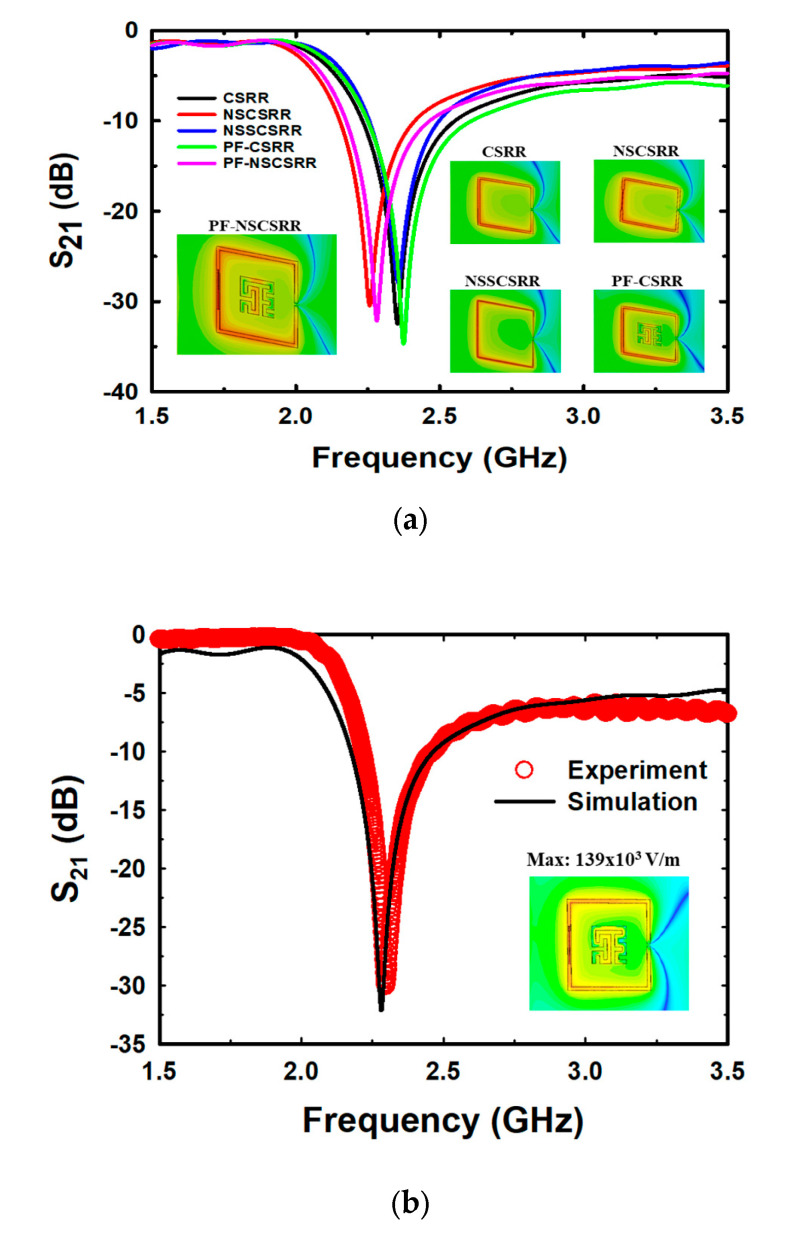
Simulation (**a**) and measurement (**b**) of the resonance frequency and electric field of the designed sensor.

The simulation results of the designed sensors are shown in Figure 5a. The CSRR at the F_r_ of 2.355 GHz has a maximum electric field of 116.8 kV/m [40], the narrow square-shaped CSRR (NSSCSRR) at the F_r_ of 2.357 GHz has a maximum electric field of 150.8 kV/m, the narrow slot CSRR (NSCSRR) at the F_r_ of 2.256 GHz has a maximum electric field of 135.3 kV/m [46], the Peano fractal geometry with CSRR (PF-CSRR) at the F_r_ of 2.374 GHz has a maximum electric field of 110 kV/m [51], and the PF-NSCSRR at the F_r_ of 2.279 GHz has a maximum electric field of 139 kV/m. These results indicate that the NSSCSRR structure provided the highest electric field while the NSCSRR provided the lowest F_r_. However, the NSSCSRR structure still operated at high frequencies while the NSCSRR structure continued to provide low electric field intensity. Considering the effect of F_r_ and electric field, the PF-NSCSRR structure was found to be the most efficient compared with all the above structures because it can generate a strong electrical field and operate at a low F_r_. Therefore, the PF-NSCSRR structure was chosen for investigation in this study. Sensors with the ability to generate high electric fields can respond rapidly to changes in MUT properties caused by changes in the ions and molecules in the sample solution [54]. Therefore, a microfluidic channel was constructed along the edge of the CSRR in the narrow slot region because it was the strongest electric field region.

Figure 5b demonstrates that the simulations and measurements of the proposed PF-NSCSRR sensor are in acceptable agreement. At the F_r_ and phase of 0°, the electric field is strongest at the narrow slot position of the lower edge of the CSRR, as shown in the insert of Figure 5b. This behavior indicates that this region is very responsive to alterations in the material properties of the MUTs. For this reason, the microfluidic channels were fabricated and placed along this region of the PF-NSCSRR sensor. The F_r_ can be seen as a notch in the S_21_ of the structure.

This research focused on designing a microwave sensor for the measurement of E2, which has a higher molecular size (a length of about 1.2 nm (1200 Å) and a width of about 0.4 nm (400 Å)), molecular weight (272.38 g/mol), and viscosity compared with water and ethanol. In order to achieve easy sample injection, low cost, and easy cleaning, a planar microwave sensor integrated with a microfluidic channel was developed while maintaining high sensitivity and compact size. For the most part, microwave sensors integrated with microfluidic channels are designed for high sensitivity and require the least amount of sample substance due to reduced microfluidic channel width and increased winding of the path, which increases the intensity of the electric field [34,40,41,42,43]. A simple structure is presented in [39]. The process is uncomplicated and requires a small sample size but has low sensitivity and operates at high frequencies. In [34,41,43], high-sensitivity structures are demonstrated. Although the procedures require a small amount of test material, their complexity and difficult fabrication increase production costs and affect the flow of the fluid sample being analyzed. In addition, cleaning is still difficult. In [42], it is capable of suppressing unwanted environmental influences but requires a large sample volume. The simplicity of the structure, ease of use, low cost, high sensitivity, compactness, ease of injection, cleanliness, and suitability of use in the measurement of samples with large molecules with higher molecular weights and viscosity compared with water and ethanol are the innovations of this work.

The initial step in the design process was to identify the optimal structures to use in developing a model, including the determination of the appropriate CSRR size, the centering area of the CSRR to be utilized in the design of the proper fractal geometry, and the fractal geometry response in the middle of the CSRR. Additionally, unlike previous research [41], which simply modified only the C_R_ value, the current study adjusted the sensor response to be sensitive and operate at low frequencies via C and C_R_ modulations.

### 2.2. Microfluidic Channel Fabrication

**Figure 6:**
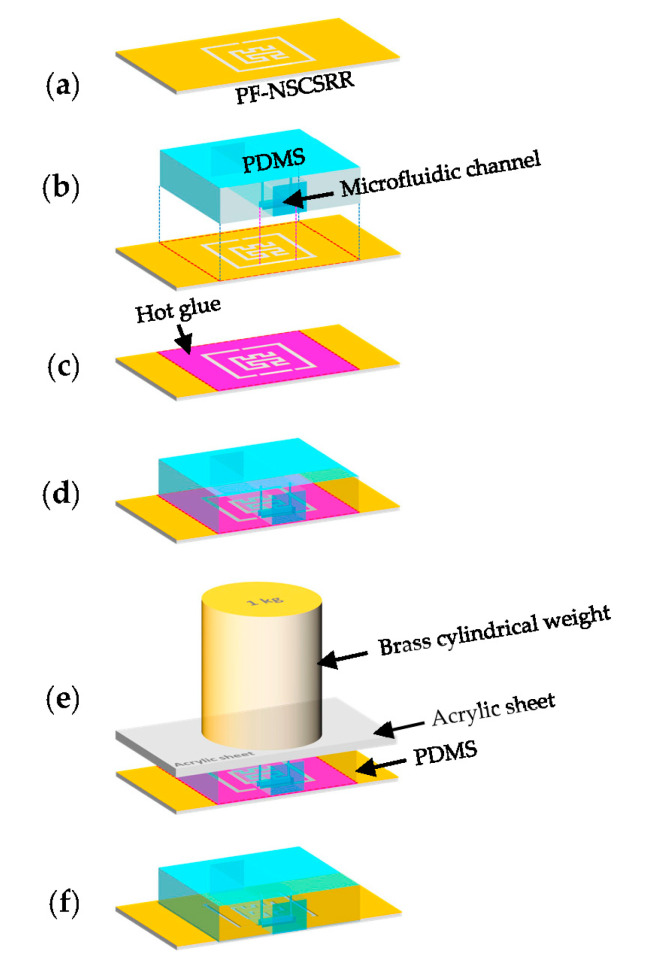
Necessary steps in connecting the PDMS to the copper surface of the sensor include (**a**) cleaning the copper surface, (**b**) attaching the PDMS to the copper surface, (**c**) covering the copper surface with hot glue, (**d**) attaching the PDMS to the copper surface, (**e**) pressing the weight on top of the device, and (**f**) testing for functionality and channel leaks.

The top of the PF-NSCSRR was covered by a PDMS membrane on which a microfluidic channel was positioned along the lower edge of the CSRR, which was the region with the narrow slot and the strongest electric field. The PDMS and reagent, in a ratio of 10:1, was used to fabricate the microfluidic channel using a mold. All air bubbles were removed using vacuum conditioning and heating for two hours at a temperature of 80 °C. The PDMS membrane was then removed from the mold and attached to the PF-NSCSRR device. The procedure for connecting PDMS to the sensor copper surface is shown in Figure 6. First, the copper surface (yellow color) of the microwave sensor was cleaned with isopropyl alcohol, then rinsed with distilled water and allowed to dry (Figure 6a). Next, the position to attach the PDMS (light blue color) to the copper surface of the microwave sensor was determined (Figure 6b). Hot glue (pink color) was applied to the sensor copper surface in the designated area and set aside for one minute (Figure 6c). The PDMS was positioned in the predetermined location on the copper surface of the sensor (Figure 6d). The acrylic sheet (gray color) was placed on top of the PDMS and the brass cylindrical weight (light yellow) was pressed against it for 30 min (Figure 6e). Finally, the glue was allowed to dry for 24 h. After that, the sensor was checked for functionality and channel leakage by injecting deionized (DI) water into the microfluidic channel (Figure 6f).

### 2.3. Preparation of Chemicals and Analyte Solutions

Analytical grade E2 (C_18_H_24_O_2_, 272.4 g/mol, purity 98.5%) and ethanol (C_2_H_6_O, 46.07 g/mol, purity ≥ 99.9%) were purchased from Sigma-Aldrich (Saint Louis, MO, USA). The E2 was dissolved in ethanol at concentrations of 0.01, 0.1, 1, and 10 mM. Due to the well-documented environmental and endocrine-disrupting effects of high E2 concentrations, we selected a range of E2 concentrations starting at 0.01 mM that can be potentially applied to environmental and clinical detection of high E2 concentrations in aquatic ecosystems, human urine, and blood samples [7,8,9,10]. E2 solutions were prepared in triplicate for each concentration.

### 2.4. Sensor Fabrication and Equipment Installation

**Figure 7:**
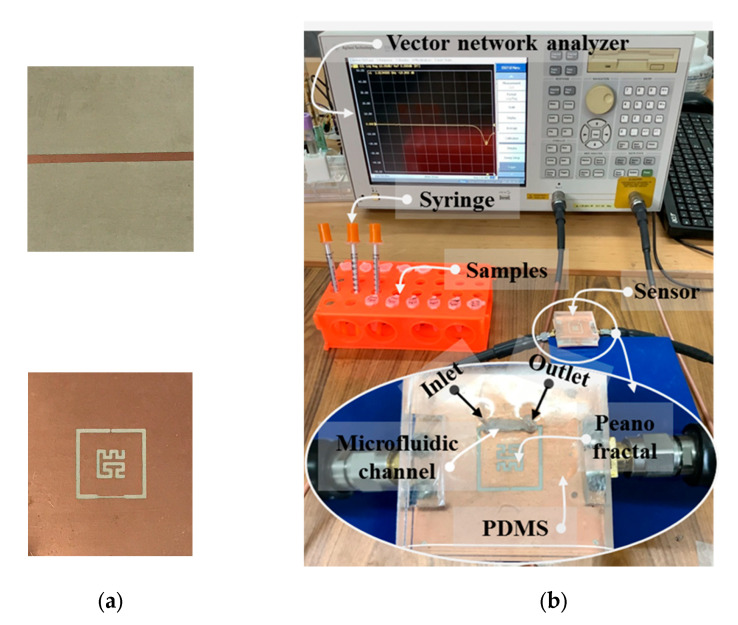
Photographs of (**a**) the fabricated PF-NSCSRR sensor and (**b**) the experimental setup.

PDMS was fitted to the area size of a PF-NSCSRR sensor and a microfluidic channel was placed over the narrow slot region. Measured responses were obtained using the Agilent E5071B vector network analyzer (VNA), calibrated, and recorded with the 1601 data points over the 0.5–2 GHz frequency range. Photographs of the fabricated PF-NSCSRR sensor are shown in Figure 7a. We fabricated the PF-NSCSRR sensor using a photolithography process. The PF-NSCSRR sensor was installed together with the microfluidic channel and connected to the VNA via a high-frequency cable (Figure 7b). To eliminate the effects of air bubbles accumulating within the microfluidic channels on the sensor response, the stop-flow technique was adopted in this study. A syringe was used to inject the liquid sample into the microfluidic channel until it was filled. Then, the injection was stopped to cease the flow so that measurements could be performed [34,35,40,41,42,43,44]. E2 was fully injected into the microfluidic channel at a volume of 1.37 μL. Each concentration of the test material was measured three times at a temperature of 27 ± 1 °C and relative humidity of 48 ± 1%. The microfluidic channel was rinsed and cleaned with DI water, and the F_r_ and S_21_ values were restored to their original values before each measurement. The MUTs tested in this work included free space, an empty channel, DI water, pure ethanol, and E2 diluted with ethanol in ten-step increments from 0.01 to 10 mM. The experiments were performed in triplicate. The data obtained from the measurements were used to calculate the mean and standard deviation.

## 3. Results and Discussion

### 3.1. Sensor Response

**Figure 8:**
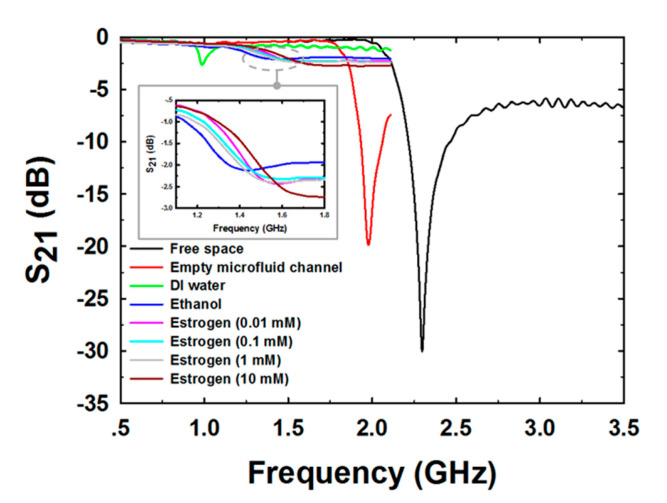
The mean magnitude of S_21_ spectra for various MUTs.

Figure 8 shows the measurement results of the S_21_ with 1601 data points covering the 0.5–3.5 GHz frequency range for free space and the 0.5–2 GHz frequency range for the empty microfluidic channel, DI water, pure ethanol, and E2 at various concentrations. In the case of measuring free space, the measured F_r_ was 2.3 GHz and the peak attention was −30.1 dB. In the case of empty microfluidic channel measurement, the measured F_r_ was reduced to 1.98 GHz and the peak attention was raised to −19.9 dB. The decrease in F_r_ was caused by an increase in capacitance generated by the setting of the PDMS microfluidic channel on the PF-NSCSRR sensor. In addition, the loss tangent of PDMS was greater than free space, which in turn increased the peak attention value. Because free space and the PDMS microfluidic channel had lower permittivity compared with DI water, the F_r_ in DI water sensing was reduced to 0.99 GHz, which was lower than the free space and the PDMS empty microfluidic channel measurements. The peak attention increased when measuring DI water (−2.66 dB) because the loss tangent increased compared with that of free space and the PDMS empty channel. In ethanol sensing, F_r_ and peak attention increased to 1.2 GHz and −1.8 dB, respectively. This is because the permittivity of the DI water is higher than that of ethanol, and the loss tangent is greater. The S_21_ spectra of the sensor response changed with changing E2 concentrations as illustrated in the gray rectangular insert of Figure 8.

**Figure 9:**
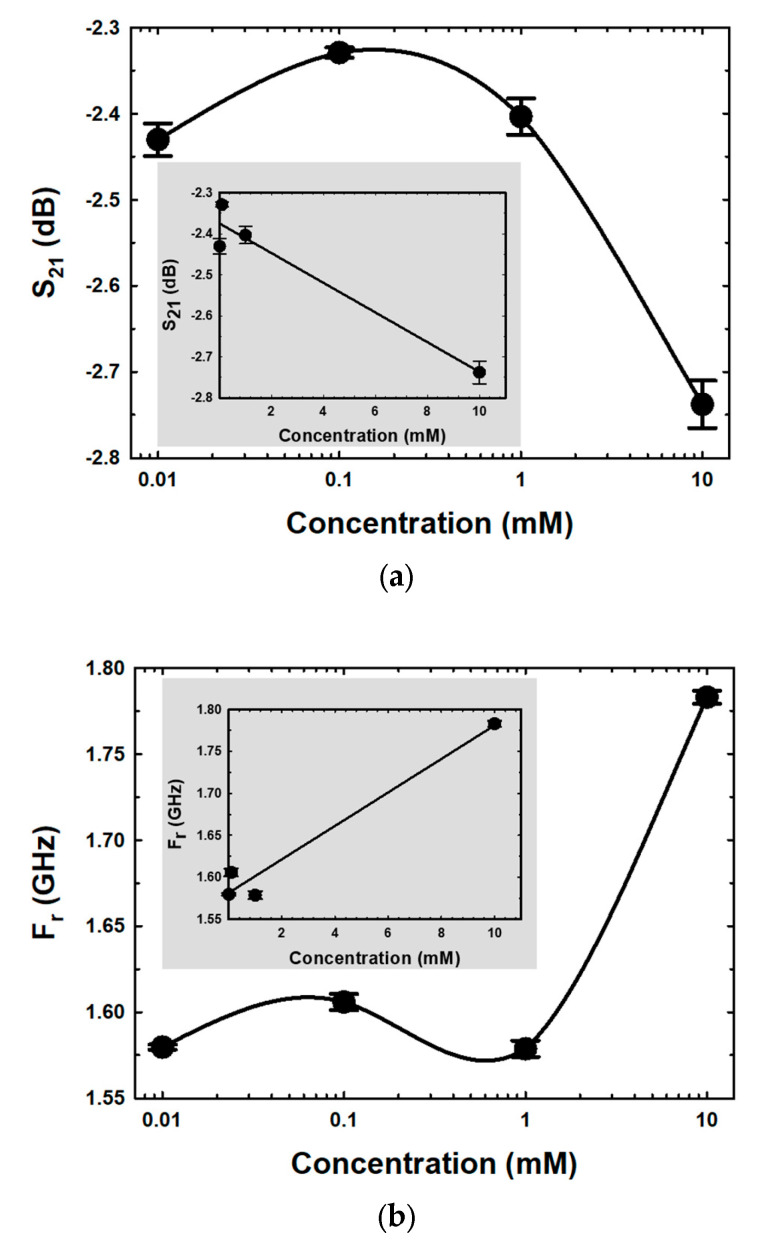
The mean magnitude of (**a**) S_21_ spectra and (**b**) F_r_ for different E2 concentrations.

In Figure 9a,b, the mean of S_21_ and F_r_, which are functions of E2 concentrations in semi-log form, are shown. The magnitude of S_21_ was raised when the E2 concentration was increased from 0.01 mM to 0.1 mM, as shown in Figure 9a. Due to the incorporation of the hydroxyl groups between ethanol and E2 molecules during growth mechanisms, the loss tangent of the MUTs increased, contributing to migration losses within the MUTs. The conductivity contribution has a significant impact on this concentration range [56]. The magnitudes of S_21_ decreased as the E2 concentration increased, whereas the peaks between 0.1 mM and 10 mM showed the opposite tendency. The loss tangent reduced as the E2 concentration increased due to the enhanced hydrogen bond interactions between the hydroxyl constituents of E2 and ethanol [57]. The self-bonding between the C-17 hydroxyl group and the C-3 hydroxyl group of E2 also increased, inducing fewer mobile dipoles in the medium. The relationship between E2 concentration and F_r_ is shown in Figure 9b. Due to the formation of hydrogen bonds between the hydroxyl group of E2, the F_r_ of resonance peaks increased when E2 concentration increased within the range 0.01–0.1 mM, resulting in less mobile dipole motion behavior of ethanol. When the E2 concentration increased, the F_r_ data for various E2 concentrations within the range of 0.1–1.0 mM decreased. This occurrence was due to the incorporation of hydroxyl groups from ethanol and E2 molecules during growth mechanisms. In addition, as the concentration of E2 increased, the dipole movement also increased. However, the F_r_ increased again when the concentration of E2 was more than 1.0 mM. This phenomenon was linked to a decrease in the free dipole of ethanol, and the larger molecular size is due to an increase in hydrogen bond interactions between the hydroxyl groups of E2, which have asymmetric and disordered molecular configurations. This behavior results in a decrease in dipole polarity, leading to a reduced dielectric constant inductance. Moreover, hydrogen bonding and solvent polarity of solvent interactions on various excited states operative in biomolecules are one cause of disordered O–H stretching vibrations of the hydroxyl groups, resulting in a change in Gibbs free energy in MUTs [57,58,59].

The inserted graphs in Figure 9a and b show the linear regression curve fittings between S_21_ and F_r_ versus the concentration of E2, respectively. The calibration sensitivity curve (slope) [60] at concentrations between 0.01 and 10 mM was determined, and the coefficient of determination (R^2^) values are displayed in Table 2.

### 3.2. Sensitivity Response

**Figure 10:**
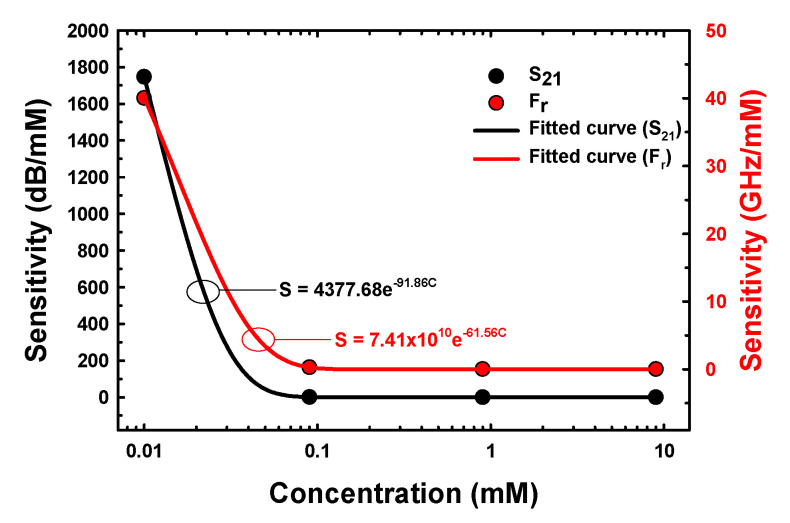
The sensitivity of the proposed sensor versus the concentrations of E2 samples.

The most important factor in a microwave sensor integrated with a microfluidic channel is sensitivity. The sensitivity (S) in S_21_ and F_r_ based microwave microfluidic sensors is defined as:

(2) $$S_{S_{21}}=\frac{{\Delta S}_{21}}{\Delta C}$$

and

(3) $$S_{F_{r}}=\frac{{\Delta F}_{r}}{\Delta C}$$

where $S_{S_{21}}$ and $S_{F_{r}}$ represent the sensitivities of the proposed microwave microfluidic sensors and C is the concentration of E2. The sensitivity of the proposed sensor with E2 in the concentration range of 0.01–10 mM based on Equations (2) and (3) is shown in Figure 10.

The sensitivity of the proposed sensor versus the concentrations of E2 samples based on S_21_ and F_r_ was a nonlinear regression within the exponential decay equations, i.e., $S_{S_{21}}=4377.68e^{-91.86C}$ and $S_{F_{r}}=7.41\times 10^{10}e^{-61.56C}$, with an R^2^ value of 1. The maximum sensitivity based on S_21_ and F_r_ at E2 concentration of 0.01 mM was 1746.98 dB/mM and 40 GHz/mM, respectively.

### 3.3. E2 Discrimination using Unsupervised Machine Learning

Since this study did not employ a specific molecule for E2 detection with the microwave sensor, the specificity of E2 detection was relatively low. Nevertheless, we hypothesized that utilizing unsupervised machine learning techniques, the data gathered from measurements of DI water, ethanol, and E2 at various concentrations can be used to distinguish between substances and categorize E2 concentrations. Analysis of the correlation between S_21_ and F_r_ with E2 concentration revealed a linear relationship that changed within a narrow range, which can distinguish differences in concentration to a certain extent. In order to distinguish the data for each E2 concentration, DI water, and ethanol more effectively, we utilized unsupervised machine learning techniques for E2 concentration classification in this study. Principal component analysis (PCA) and K-means clustering were taken as two powerful techniques for unsupervised machine learning that enabled data analysis without any predefined target or outcome. In this context, PCA was used to reduce the dimensionality of the data by identifying and extracting the most important features that capture the most variation in the data. On the other hand, K-means clustering was utilized to group similar data points into clusters based on their similarity or distance from each other. Several disciplines, including finance, biology, image and signal processing, and natural language processing, among others, make extensive use of PCA and K-means clustering.

**Figure 11:**
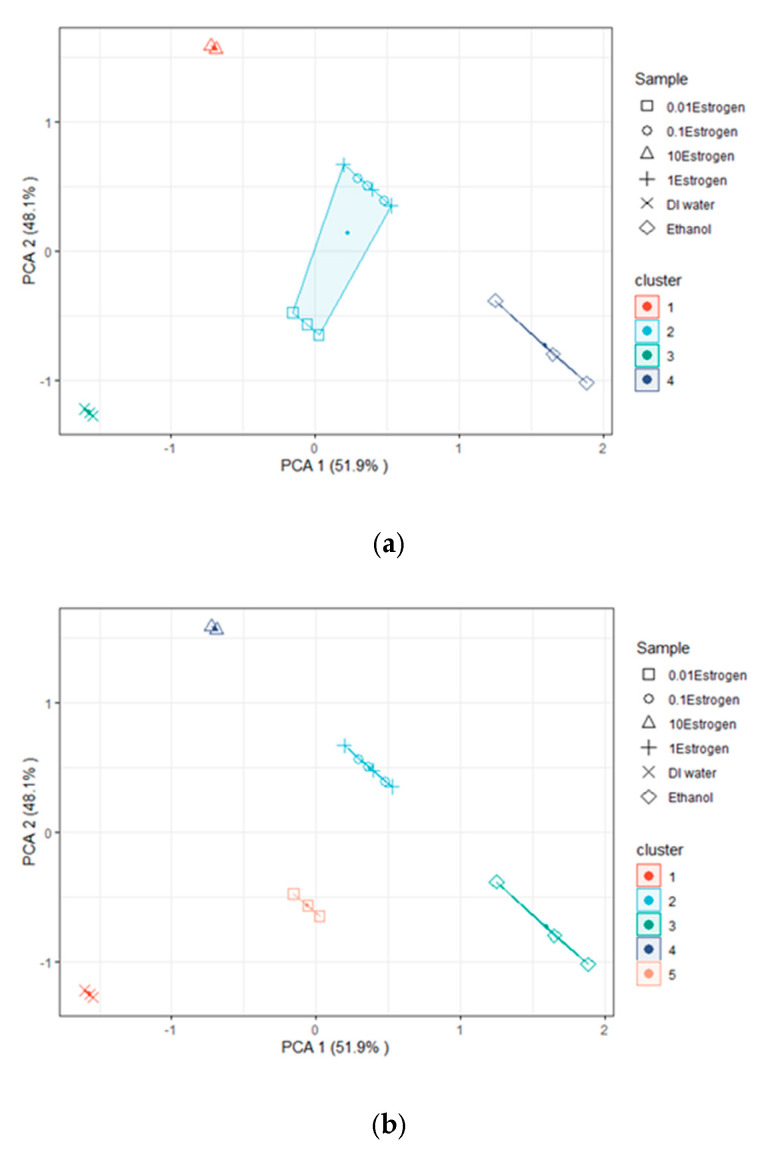
Clustering with (**a**) K = 4 and (**b**) K = 5 clusters.

PCA with a K-mean clustering algorithm was applied to classify S_21_ and F_r_ data received from various MUT measurements. The data were recorded in triplicate and imported into Microsoft Excel^®^ 2010. The data for each sample were averaged and the standard deviation was calculated for each repeated measurement in each sample. The data points that made up the cluster were defined using PCA and K-mean clustering. K-means clustering was performed using the factoextra R Package, with four and five clusters (K = 4 and K = 5). Figure 11a shows four clusters (K = 4) of five MUTs consisting of DI water, ethanol, and E2 at concentrations ranging from 0.01 mM to 10 mM, which were increased 10X at each step (without free space and an empty microfluidic channel). The S_21_ and F_r_ acquired from various MUT measurements were analyzed using a PC with 6 variables and 36 data points. According to the results, the proposed sensors were shown to be capable of detecting and distinguishing MUTs into four categories, including DI water, ethanol, 10 mM E2, and E2 at concentrations in the range of 0.01–1 mM. The results revealed that the quadrant clearly separated the DI water, ethanol, and 10 mM E2. There was an overlap between quadrants when E2 doses ranged from 0.01 to 1 mM. However, when five clusters (K = 5) were used, it was found that the quadrants separated DI water, ethanol, 10 mM E2, and E2 at concentrations of 0.1 mM and 1 mM (the 0.1 mM and 1 mM concentrations overlapped), as shown in Figure 11b. The results showed that 0.01 mM E2 was an overlap between quadrants that separated DI water and ethanol.

### 3.4. S_21_ and F_r_ Analysis

**Figure 12:**
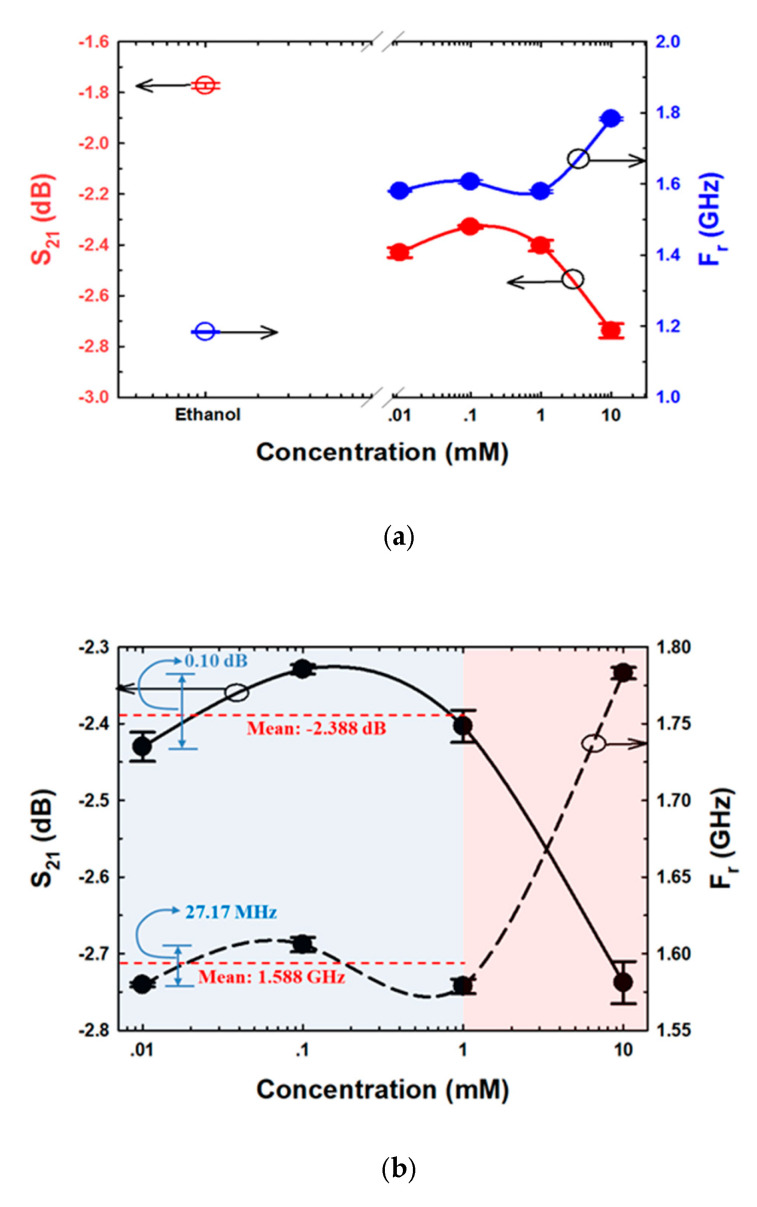
S_21_ and F_r_ sensing of (**a**) ethanol and various E2 concentrations, and (**b**) different E2 concentrations.

**Figure 13:**
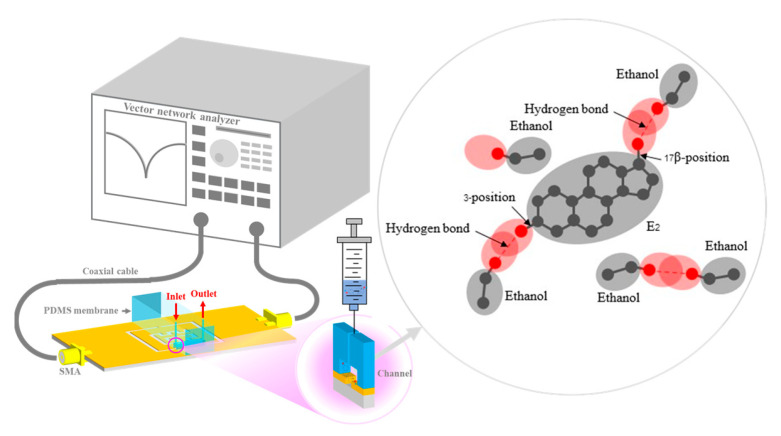
The schematic and mechanism of E2 concentration sensing.

The S_21_ and F_r_ data obtained from ethanol and various E2 concentration measurements are shown in Figure 12. As demonstrated in Figure 12a, the S_21_ and F_r_ parameters of ethanol and 10 mM E2 differed considerably from those of 0.01 mM and 1 mM E2. In addition, compared with the other E2 concentrations, the S_21_ and F_r_ levels of ethanol and 0.01 mM E2 were the most similar to each other. The results are in line with the results of culturing, as shown in Figure 11a,b. Because the S_21_ and F_r_ levels of the range of E2 concentrations were close, as demonstrated in Figure 12b, 0.01–1 mM E2 concentrations were grouped as shown in Figure 11a. The means of S_21_ and F_r_ were −2.388 dB and 1.588 GHz, respectively. The differences in S_21_ and F_r_ levels were 0.1 dB and 27.17 MHz, respectively. The limit of detection (LOD) is calculated based on the standard error of the response on the slope of the calibration curve as LOD = 3(S_yx_/m), where S_yx_ and m are the standard error and the slope of the calibration curve, respectively [61]. The results showed that LOD calculated based on S_21_ and F_r_ were 4.355 mM and 3.4 mM, respectively. The ligand binding and dipole moment affect the signal pattern measured [62,63,64]. Because ethanol has a higher dielectric constant (ε = 25.02) [65] than E2 (ε = 2) [66], the F_r_ obtained from ethanol was lower than the F_r_ obtained from 0.01 mM E2 (Figure 12a). The main intermolecular attraction in pure E2 and ethanol molecules is hydrogen bonding [59]. After mixing E2 with ethanol, the hydrogen bonds that existed between the molecules of these two pure substances were broken. When the molecules are combined, new hydrogen bonds were formed between E2 molecules and ethanol molecules based on the “like-dissolves-like” rule [67], as shown in Figure 13. E2 and ethanol are polar molecules with their dipole moments close to each other. E2 is soluble in ethanol. The dipole moment data for ethanol and E2 are 1.7D [65] and 2.2D [68], respectively.

The orientation of the OH groups, breaking of H-bonding, forming of H-bonding, and the thermal reorganizations of the E2 and ethanol molecules induced the change in dielectric constant and dielectric loss [69], which led to the change in S_21_ and F_r_, as shown in Figure 12.

### 3.5. Performance Comparison of the Microwave Sensor

Table 3 compares information about the proposed sensor for E2 to that of other sensors. In comparison to the recent aptamer-based analytical methods, the proposed method required a smaller test volume. In addition, the results demonstrated that the proposed microwave sensor was capable of measuring E2 over a wide and very high concentration range. In contrast, previous studies measured E2 concentrations within the range of 0–0.370 mM [16,17,18,19,20]. References [16,18,19] used the μM scale (the specimen volumes are 500 μL, >100 μL, and 160 μL, respectively), reference [14,17] used nM levels (the specimen volume of reference [14] was 10 μL, but the data for reference [17] were not available), and reference [15] and reference [20] used pM levels (the specimen volume data were not available).

Table 4 shows a comparison of the performance of the previous sensors with the proposed sensor design. The microwave sensors integrated with microfluidic channels in references [30,34,37,38,39,40,41,42,43] were evaluated using ethanol and methanol specimens, whereas the sensor devices in references [44,45,46] were tested using glucose specimen. The structures of representative sensors were divided into four groups, including CPW resonator [30], SRR [38], CSRR [34,37,39,40,42,43,44], and CSRR modified with another method [41,45,46]. All of the above-mentioned methods identified the properties of MUTs based on the changes in F_r_ and the magnitudes of S_11_ and S_21_.

In [40], CSRR devices and microfluidic channels are designed for dielectric characterization. However, this device still operates at a high frequency and has a low-quality factor (Q factor). The CSRR structure is improved for increased sensitivity and Q factor by optimizing the resonator using pixelization and shape optimization [52] and adjusting the CSRR slot to be narrower in some areas [54]. However, both methods still use a large sample volume and operate at a high frequency. For increased sensitivity and decreased sample volume through integration of a microfluidic channel for EQ sensing that can satisfactorily measure the EQ, the Peano fractal geometry with CSRR (PF-CSRR) is proposed in [51]. However, the PF-CSRR still operates at high frequencies, with low sensitivity and Q factor, and requires a large sample volume. Moreover, it still has low detection potential, especially for endogenous estrogenic hormones. Therefore, the PF-NSCSRR structure has been presented in the current work for superior E2 measurement performance, as it can operate at low frequencies with increased sensitivity and Q factor. It combines the strengths of the NSCSRR (high Q factor) and PF-CSRR (high sensitivity (S_Fr_)) structures. The CSRR slot was made to be narrower in some areas and the Peano fractal geometry was added to the middle of the CSRR structure in the ground plane, as illustrated in Figure 1a. To further aid in lowering the operating frequency and increasing the Q factor, the substrate material has also been changed to one with higher dielectric value and lower thickness compared with the structure presented in [51]. In addition, in order to reduce the sample volumes and increase the electric field intensity in the sensing area, the slot size of the CSRR and the microfluidic channel were decreased. In comparison to the original PF-CSRR sensor [51], the proposed sensor sensitivity and quality factor were increased by 6.08% and 40.72%, respectively. In addition, the operating frequency, active area, and sample value of the proposed sensor were decreased by 1.71%, 25%, and 28.27%, respectively. The improved properties of the proposed structure in comparison to the original sensor and previously proposed structures for detecting completely similar samples are shown in Table 5.

## 4. Conclusions

This study investigated and proposed a method for detecting E2 at high concentrations using a microwave sensor integrated with a microfluidic channel. The results revealed that the proposed method can effectively reduce the sample volume, simplify protocols compared with traditional immune-based assays, and enhance the sensitivity, quality factor, operating frequency, and active area of the sensor. The values of maximum sensitivity, as indicated by S_21_ and F_r_, were 1746.98 dB/mM and 40 GHz/mM, respectively. The channel area and sample volumes were 2.7 mm^2^ and 1.37 μL, respectively. The magnitudes of S_21_ and F_r_ were analyzed to determine their relationships to the concentration of E2 in a logarithmic and linear-concentration manner. LOD calculated based on S_21_ and F_r_ were 4.355 mM and 3.4 mM, respectively. PCA and K-means clustering (K = 4 and K = 5) were used to analyze and group the samples. The proposed method can detect E2 with high sensitivity, even at concentrations in the range of 3.4–10 mM. The results of the PCA and K-means analyses suggest that unsupervised machine learning techniques can be used to classify and group samples based on E2 concentrations. This can be useful in identifying the presence of and quantifying E2 in a sample. The implications of these findings can be significant for fields related to E2 detection, such as environmental monitoring and medical diagnostics. The proposed method can provide a more efficient and accurate way of detecting E2, which can have important implications for human health, ecologically relevant animal species, and the environment. Additionally, unsupervised machine learning techniques can provide a more comprehensive analysis of the data, which can help researchers better understand the relationship between E2 concentration and sensor output.

In the future, our team intends to develop microwave sensors capable of measuring E2 over a wider range of concentrations, spanning both in vivo and environmental concentrations. Additionally, we will explore various artificial intelligence (AI) techniques to further improve the accuracy and specificity of E2 detection. This research has the potential to revolutionize E2 detection and has significant implications for the fields of medicine and environmental science.

## Figures and Tables

**Table 1:** Geometrical parameters of the PF-NSCSRR device.

Parameter	W	L	c	s	l	g
Value (mm)	1.3	11	0.5	0.2	4.5	0.2

**Table 2:** Regression equations between concentrations of E2, transmission coefficient, and resonance frequency.

Dependent Variables	Regression Equations	R^2^
S_21_	S_21_ = −0.0362C − 2.3746	0.9433
F_r_	F_r_ = 1.96 × 10^6^C + 1.58 × 10^9^	0.9646

C: concentration (mM).

**Table 3:** Comparison of different methods of E2 sensing.

Ref.	Sensors	Spec.	Conc.	Data	Volume (µL)	LOD	Linear Range	Time Required
[14]	Electro-chemical aptasensor	E2, milk, serum	0–15 nM	Δ*I*	10	0.5 pM	1.5–100 pM and 100–7000 pM	~30 min
[15]	Aptasensor based on pNIPAm microgel-based etalons	E2, milk	0–734 pM	Δ*λ*	NA	3.2 pM	3.2–640 pM	~30 min
[16]	Colorimetric aptasensor based on gold nanoparticle with DNA aptamer	E2	0–4.5 µM	Δ*A*	500	1.57 nM	1.57–350 nM	~30 min
[17]	Fluorescent aptasensor based on Ru complex and quantum dots	ADE, DA, E2	0–400 nM	Δ*F*	NA	37 nM	0.08–0.4 µM	~1 h
[18]	Fluorescent aptasensor based on gold nanoparticle and RhoB	E2	0–1.5 µM	Δ*F*	>100	0.48 nM	0.48–200 nM	~30 min
[19]	Colorimetric aptasensor based on DNA aptamer and gold nanoparticle	E2	0–370 µM	Δ*A*	160	~367 pM	~0.367–367,000 nM	~35 min
[20]	PEC and DNA aptamer and TiO_2_-BiVO_4_	E2	0–250 pM	Δ*I*	NA	22 fM	0.1–250 pM	>1 h
TW	PF-NSCSRR	Free space, DI water, Ethanol, E2	0–10 mM	F_r_, S_21_	1.37	3.4 mM	0.001–10 mM	<1 min

NA: data not available; Conc.: concentration; TW: this work.

**Table 4:** Comparison of integrated microwave sensors with microfluidic channels.

Ref.	Structure	Spec.	Conc.	Meth.	F_r_ (GHz)	Sensitivity	Volume (μL)	Channel Area (mm^2^)
[30]	CPW resonator	Ethanol	0–20% by volume	F_r_, S_21_	20	−63.1 MHz; 3.56 × 10^−3^	2.95 × 10^−3^	0.074
[34]	Series LC	Ethanol	0–100% by volume	F_r_, S_21_	1.91	0.45%	0.637	9.1
[37]	CSRR	Ethanol	0–100% by volume	F_r_	4.72	49.1 MHz	3	5
[38]	SRR	Ethanol, Methanol	0–100% by volume	F_r_	2.1	NA	0.108	1.8
[39]	CSRR	Ethanol	0–100% by volume	F_r_	2.3	NA	236	0.79
[40]	CSRR	Ethanol	0–100% by volume	F_r_	2.02	0.308%	0.588	0.042
[41]	Series LC	Ethanol	0–100% by volume	F_r_	1.662	0.695%	0.7	0.02
[42]	MCSRR	Ethanol	0–100% by volume	F_r_	1.62	0.469%	5.4	0.2
[43]	CSRR	Ethanol	0–100% by volume	F_r_	2.226	0.62%	0.52	0.02
[44]	CSRR	Glucose	0–80 mg/mL	F_r_	2.48	0.5 × 10^−3^ MHz and 0.5 dB	0.637	9.1
[45]	TP-CSRR	Glucose	0.7–1.2 mg/mL	S_21_, S_11_	2.3	1.7–6.2 dB (S_21_) and 0.6–3.45 dB (S_11_)	648	324
[46]	CSRR-interferometric system	Glucose	0–400 mg/dL	F_r_, S_21_	2.26	1.947 mdB	6.9	11.06
TW	PF-NSCSRR	Ethanol, E2	0–10 mM	F_r_, S_21_	2.30	40 GHz (F_r_) and 1746.98 dB (S_21_)	1.37	2.7

Spec.: Specimen; Conc.: Concentration range; Meth: Method; NA: Data not available.

**Table 5:** Comparison of the microwave sensors with previous designs.

Ref.	Structure	Spec.	Conc.	Meth.	F_r_ (GHz)	Sensitivity	Q-Factor	Channel Area (mm^2^)	Volume (μL)
[51]	PF-CSRR	DMSO, EQ	0–100 mM	F_r_, S_21_	2.34	61.97 GHz (F_r_) and 1646.87 dB (S_21_)	81.64	3.6	1.91
[52]	Pixelated pattern resonator	Cyclohexane (C_6_H_12_), Chloroform (CHC_l3_)	0–100%	F_r_, S_21_	5.64	NA	NA	104.04	NA
[54]	SCSRR	Phosphate, Nitrate	0–1 mg/mL	F_r_, S_21_	2.33	241 MHz (F_r_) and 3.21 dB (S_21_)	95.00	12.57	100
TW	PF-NSCSRR	Ethanol, E2	0–10 mM	F_r_, S_21_	2.30	40 GHz (F_r_) and 1746.98 dB (S_21_)	114.88	2.7	1.37

Spec.: Specimen; Conc.: Concentration range; Meth.: Method; NA: Data not available.

## Data Availability

Not applicable.
